# Evaluating fumonisin contamination in cattle feed: Impact on animal health, the agriculture industry and regulatory considerations

**DOI:** 10.1016/j.crtox.2025.100235

**Published:** 2025-04-26

**Authors:** Ashli A. Brown, Tim Herrman

**Affiliations:** a Office of the Texas State Chemist, Texas A&M AgriLife Research, Texas A&M University System, College Station, TX 77841, United States; b Department of Soil and Crop Sciences, Interdisciplinary Faculty of Toxicology, Texas A&M University, College Station, TX 77843, United States

**Keywords:** Fumonisin, Guidance levels, Beef cattle, Dose–response, Ruminants, Fusarium contamination

## Abstract

In 2001, the US FDA released guidance levels (GLs) for fumonisin (FUM) in corn and corn by-products intended for human and animal consumption. Recent research challenges the conservatism of these GLs for ruminants, particularly for regions like the Texas High Plains, where blending permissions have been provoked, limiting the management of FUM contamination economically and maximization of corn availability for feedlots. This study evaluates whether scientific advancements since 2001 warrant a revision of these guidelines. Specifically, a 2020 study provides new data on cattle fed FUM concentrations up to 108.8 mg/kg, suggesting the current 60 mg/kg GL may be overly conservative. Using a combination of *meta*-analysis and the US EPA’s Benchmark Dose Software (BMDS), we assess the new scientific evidence. Our findings indicate that new data does not significantly refine the dose–response relationship for FUM in cattle, supporting the continued appropriateness of the current 60 mg/kg GL.

## Highlights

• Current regulatory guidance for fumonisin in ruminant rations is based on limited studies conducted before 2001.
• A 2020 study found that cattle fed above the recommended level for 110 days did not show significant adverse effects.
• This paper explores refining fumonisin guidance using a comparative approach of a *meta*-analysis vs. benchmark dose modeling.

## 1 Introduction

Fumonisins (FUMs) are secondary metabolites produced by pathogenic molds of the *Fusarium* spp. that infest corn globally (Food and Administration, 2001a, Kamle et al., 2019). Of the several FUM analogs, fumonisin B_1_ (FB_1_) is the most prevalent, toxic, and extensively studied member (Food and Administration, 2001a, Voss et al., 2007). Other forms, including fumonisin B_2_ (FB_2_) and fumonisin B_3_ (FB_3_), are also naturally occurring but generally less prevalent (Food and Administration, 2001a, Voss et al., 2007). Toxicological evidence identify FUMs as potent disrupters of sphingolipid homeostasis that have been associated with esophageal cancer in humans, neural tube defects in neonates, and several systemic effects in animals (Kamle et al., 2019).

Consequently, the US FDA issued a guidance document in 2001 providing direction for managing total FUMs (FB_1_ + FB_2_ + FB_3_) in corn and corn-based products intended for human and animal consumption with good manufacturing and agricultural practices (FDA, 2001a). As shown in Table 1, some GLs are based on limited studies or insufficient data for low FUM exposure in certain animal species (FDA, 2001b). Notably, the GL for cattle was set at 60 mg/kg (with a limit of 50 % of the diet on a dry matter basis), primarily selected based on Osweiler et al. (1993), which reported mild liver and immune system effects in calves fed 110.3 mg/kg of FUM for 31 days. Missing data on ruminants exposed to intermediate doses (25 to 100 mg/kg) led to the selection of a more conservative GL, 60 mg/kg, under the assumption that higher levels may lead to adverse health effects such as reduced feed and water intake, hepatotoxicity, immunotoxicity, and pulmonary edema (Food and Administration, 2001b, Baker and Rottinghaus, 1999, Osweiler et al., 1993, Smith and Thakur, 1996).

**Table 1:** Summary of pivotal peer-reviewed studies used as basis of the current FUM guidance.

Animal or class	No. of pivotal studies	Average year of publication	
Equids	18	1992	
Rabbits	2	1997	
Swine	24	1995	
Catfish	4	1995	
Rainbow trout	1	1998	
Ruminants (cattle, sheep, goats)	5	1996	
Mink	3	1996	
Poultry (turkeys, chickens, and ducklings)	23	1995	
Rats and mice	1	1999	
Domesticated species (cats and dogs)	−	−	

Since the release of the 2001 GL, Jennings et al. (2020) have demonstrated that cattle fed up to 108.8 mg/kg FUM for roughly 110 days did not exhibit significant adverse health effects. This finding challenges the current 60 mg/kg, particularly for perennial hotspots of mycotoxin contamination, such as Texas High Plains that have experienced FUM levels more than three times greater than the 60 mg/kg GL and are no longer authorized to maximize corn availability for feedlots and manage FUM contamination economically through blending permissions (Brown et al., 2024, Herrman et al., 2018). This paper reviews recent advancements in FUM research related to cattle and evaluates their implications for the current GL, using *meta*-analysis and the EPA's Benchmark Dose Software (BMDS) to assess whether a revision of the GL is necessary.

## 2 Materials and methods

We conducted a literature search in the PubMed database using the following search terms: '(((((beef) OR (cattle)) OR (feedlot)) AND (fumonisin)) OR (FB_1_)) OR (fusarium).' Studies were screened using the PECO (Population, Exposure, Comparator, and Outcomes) criteria outlined in Table 2. We excluded studies that did not meet our criteria, investigated alternate exposure routes, or involved multiple contaminants due to differences in bioavailability and mode of action. Selected studies were used to compare acceptable levels derived from *meta*-analysis with those generated by BMDS to assess the impact of recent data on the 60 mg/kg FUM GL for cattle.

**Table 2:** PECO statement used in the systematic lit search.

**Element**	**Inclusion criteria**
**P**opulation	Cattle
**E**xposure	Total dietary fumonisin (FB_1_ + FB_2_ + FB_3_) with restrictions on oral administration
**C**omparison	A comparison of cattle exposed to various levels of fumonisin; experimental studies include at least one control group and one treatment group exposed to fumonisin only
**O**utcome	Adverse effects on cattle performance^a^, liver, and any other associated health outcomes^b^

a Adverse outcomes on cattle performance include body weight, body condition, feed intake, etc.

b Other associated health outcomes may include those effects associated with cell autophagy and apoptosis, neurotoxicity, immunotoxicity, and tissue and organ toxicity.

## 3 Results

### 3.1 Literature review

Table 3 summarizes the four experimental feeding studies identified in our literature search. Studies involving FUM toxicity through alternate routes of administration or examining FUM combined with other mycotoxins were excluded due to differences in bioavailability and mechanisms of action (Albonico et al., 2016, Awapak et al., 2021, Mathur et al., 2001, Roberts et al., 2021, Wang et al., 2020). Three of the selected studies were available when the US FDA released the current GLs for cattle; however, two studies did not evaluate FUM concentrations close to 60 mg/kg GL, making them unsuitable for assessing a safety breakpoint. Therefore, only two studies—Osweiler et al. (1993) and Jennings et al. (2020)—met the necessary criteria for comparison.

**Table 3:** Overview of available literature for experimental cattle exposed to dietary FUM.

Reference	No. of cattle (dose groups)	Body weight (BW, kg)	Exposure duration (days)	Average FUM content: FB_1_ + FB_2_ + FB_3_, (mg FUM/kg BW)^a^	Employed toxicological tests	Statistically significant findings associated with FUM
*Studies used as basis of current US FDA ML for fumonisin*						
Osweiler et al. (1993)	18 (3)	231 ± 5.7	31	• Control: < 0.13	• Feed intake	• Liver function tests
					• Weight gain	
				• Treatment 1: 0.78	• Hematology test	• Neutrophil function
				• Treatment 2: 3.7	• Liver function test	• Lymphocyte blastogenesis
					• Neutrophil function test	
					• Lymphocyte blastogenesis	
					• Histopathological analysis	
Smith & Thakur (1996)^b^	6 (2)	217	30	• Control: < 0.13 • Treatment: 5.15	• Liver function test	• Liver function tests
					• Organ-to-BW ratio • Feed-to-tissue ratio • Histopathological analysis	
Baker & Rottinghaus (1999)^b^	5 (2)	86 to 127	239 to 253	• Control: < 0.13 • Treatment: 11	• Liver function test • Biomarker analysis • Histopathological analysis • Hematology test	• Liver function tests
*Literature advances since 2001*						
Jennings et al. (2020)	50 (5)	361 ± 6.4	110	• Control: 0.2 • Treatment 1: 0.67 • Treatment 2: 1.02 • Treatment 3: 1.69 • Treatment 4: 2.72	• Feed intake • Performace test • Weight gain • Biomarker analysis • Carcass grading & evaluation • Histopathological analysis & scoring	• Biomarker analysis

a Average dietary FUM concentration is calculated with the assumption that experimental cattle consumed 2.5% of BW per day.

b Studies did not investigate FUM concentrations proximate to the present regulatory limit of 0.75 mg/kg BW/d and were excluded.

Osweiler et al. (1993) evaluated the effects of control (≤5 mg/kg), low (31 mg/kg), or high (148 mg/kg) FUM diets fed at 2 % BW (body weight) on cattle health for 31 days through performance tests, hematology, liver and neutrophil function, lymphocyte blastogenesis, and histopathological analyses. Jennings et al. (2020) investigated the health effects of FUM levels ranging from 8.1 to 108.8 mg/kg for 110 days at 2 % BW through performance tests, biomarker analyses, carcass grading, histopathology scoring, and FB_1_ biomarker analyses, including the ratio of sphinganine (SA) to sphingosine (SO) (SA:SO). Both studies reported mild to moderate changes in liver enzyme activity for high-dose groups, but these changes did not significantly impact animal performance at any of the tested levels. Additional observations from Osweiler et al. (1993) included impaired immune function, reduced feed intake, and slow weight gain. Jennings et al. (2020) did not investigate immunological changes but noted a slight increase in BWs as FUM contamination increased.

### 3.2 Comparative analysis

Studies deemed suitable for comparison exhibited insufficient overlap in the endpoints assessed, precluding a meaningful *meta*-analysis. Osweiler et al. (1993) measured feed intake, BW, bilirubin, cholesterol, and liver enzymes, aspartate transferase (AST), gamma-glutamyl transferase (GGT), and lactate dehydrogenase (LDH). In contrast, Jennings et al. (2020) used a histopathology scoring system to document FUM-induced liver, kidney, and skeletal damage, along with carcass characteristics, BW, and ratios of FB_1_ biomarkers in liver samples. The heterogeneity in BW trends across the studies further hindered the comparison of scientific data.

**Fig. 1:**
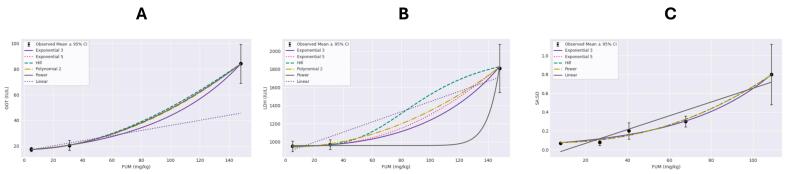
BMD analyses at 10% RD of dietary FUM on GGT (A) and LDH (B) enzymes reported by Osweiler et al. (1993) and SO:SA levels (C) reported by Jennings et al. (2020).

We evaluated the hepatic endpoints reported by Osweiler et al. (1993) and Jennings et al. (2020) using continuous model types in the BMDS with specific parameters on normal distribution and non-constant variance, respectively. The BMD analyses were run at the most common Benchmark Response (BMR) values of 1 standard deviation of 1 and 10 % relative deviation (RD), providing six possible modeling options for each dataset (e.g., Exponential 3, Exponential 5, Hill, Linear, Polynomial, and Power). Changes in data deviation types did not affect results; thus, we only show findings generated at 10 % RD. Table 4 presents the BMDS recommendations and notes for all modeling options, except the Polynomial model, as the software does not provide recommendations for this option due to concerns related to its reliability in risk assessment. Fig. 1 shows the observed mean response at a 95 % confidence interval against increasing FUM doses with multiple BMD model possibilities. The AST liver enzyme and SA:SO ratios were the most complicated endpoints to model as all options were deemed questionable or unusable, likely caused by high response variability in the high FUM dose groups, indicating the EPA's BMDS was unable to fit the data adequately. More specifically, modeling options for AST could not be graphed due to the extremely poor fitting. Common issues across modeling options for AST and SA:SO ratios included failed non-constant variance tests, poor goodness-of-fit, and zero degrees of freedom. For GGT and LDH enzymes, Exponential 3 was the best fitting model based on the lowest Akaike Information Criterion (AIC). However, the 18.2 mg/kg BMD value generated for GGT falls below the current 60 mg/kg GL, revealing its irrelevancy in refining the current GL. On the other hand, the 64.3 mg/kg BMD value generated for LDH supports the current 60 mg/kg GL.

**Table 4:** The BMD recommendations modeling hepatic endpoints for Osweiler et al. (1993) and Jennings et al. (2020).

						**BMDS Recommendations and Notes**				
	**Study**	**FUM dataset**	**Endpoint**	**Response means**	**Response variability**	**Exponential 3**	**Exponential 5**	**Hill**	**Linear**	**Power**
	Osweiler et al. (1993)	5, 31, and 148	AST	70.5, 79.5, 392.3*	2.1, 10.1, 136.7*	Unusable	Questionable	Questionable	Questionable	Questionable
						Did not successfully execute.	Zero degrees of freedom; saturated model	Zero degrees of freedom; saturated model	Nonconstant variance test failed (Test 3p-value < 0.05)	Zero degrees of freedom; saturated model
							Nonconstant variance test failed (Test 3p-value < 0.05)	Nonconstant variance test failed (Test 3p-value < 0.05)	Goodness of fit p-value < 0.1	Nonconstant variance test failed (Test 3p-value < 0.05)
							Control stdev. fit > 1.5	Control stdev. fit > 1.5	Control stdev. fit > 1.5	Control stdev. fit > 1.5
	Osweiler et al. (1993)	5, 31, and 148	GGT	17.5, 20.5, 84.3*	1.71, 3.67, 14.45*	Recommended: lowest AIC	Questionable	Questionable	Questionable	Questionable
						BMD − 18.2	Zero degrees of freedom; saturated model	Zero degrees of freedom; saturated model	Zero degrees of freedom; saturated model	Goodness of fit p-value < 0.1
						BMDL − 8.2				
						BMDU − 41.3				
	Osweiler et al. (1993)	5, 31, and 148	LDH	952.8, 970.7, 1811.1*	55.9, 51.3, 255.8*	Recommended: lowest AIC	Questionable	Questionable	Questionable	Questionable
						BMD − 64.3	Zero degrees of freedom; saturated model	Zero degrees of freedom; saturated model	Zero degrees of freedom; saturated model	Goodness of fit p-value < 0.2
						BMDL − 34.7				
						BMDU − 134.5				
	Jennings et al. (2020)	5.0, 26.7, 40.7,	SA:SO	0.07, 0.08, 0.2,	0.01, 0.05, 0.12,	Questionable	Questionable	Unusable	Unusable	Unusable
		67.7, and 108.8		0.3, and 0.8*	0.08, and 0.45*	Control stdev. fit > 1.5	Lowest dose/BMDL ratio > 3	Control stdev. fit > 1.5	Lowest dose/BMD ratio > 3	Control stdev. fit > 1.5
						Constant variance test failed (Test 2p-value < 0.05)	Lowest dose/BMDL ratio > 10	Constant variance test failed (Test 2p-value < 0.05)	Lowest dose/BMD ratio > 10	Constant variance test failed (Test 2p-value < 0.05)
							Control stdev. fit > 1.5	BMDL does not exist	Goodness of fit p-value < 0.1	BMDL does not exist
							Constant variance test failed (Test 2p-value < 0.05)		Control stdev. fit > 1.5	
							BMD/BMDL ratio > 3		Constant variance test failed (Test 2p-value < 0.05)	
							BMD/BMDL ratio > 20		BMDL does not exist	

*Reported to differ from corresponding control.

## 4 Discussion

In this paper, we determine whether the recent advancements in experimental data for fumonisin in cattle, as published by Jennings et al. (2020) influence the current regulatory GL of 60 mg/kg using *meta*-analysis and the EPA's BMDS. While Jennings et al. (2020) provided valuable data, a meaningful *meta*-analysis was impossible due to the lack of overlap in measured hepatic endpoints and the unclear BW trends with Osweiler et al. (1993). Likewise, although dose–response relationships for both datasets showed monotonic trends, significant inter-individual variability in responses of liver enzymes and FB_1_ biomarkers for high-dose groups limited BMDS’s ability to capture an effective toxicological response pattern. We identified a BMD of 64.3 mg/kg for the liver enzyme, LDH, aligning with the current GL. However, we were unable to substantiate this value with insight from SA:SO ratios reported in the most recently published feedlot study, as we aimed. The key differences in study designs and their potential impact on the response variability emphasize how these inconsistencies can create difficulty in refining regulatory guidelines.

The impacts of FUM contamination extend deep, particularly affecting cattle health and farmers reliant on harvesting crops primarily for livestock feed in areas most susceptible to FUM contamination, like the Texas High Plains (Brown et al., 2024). In recent efforts to help address such concerns, Qu et al. (2022) have shed light on the advantages of biodetoxification methods in mitigating mycotoxin contamination in agricultural products. At a molecular level, Chen et al. (2021) have broadened the understanding of FB_1_ disruption of cellular functions and its critical roles in signaling pathways and sphingolipid metabolism.

Further advancements include the availability of bioactivity data through online tools such as the EPA’s Computational Toxicology (CompTox) Chemicals Dashboard, which has provided insights into the FB_1_ interactions in single and multicell mutagenicity assays, as well as its carcinogenicity potential in rodent models. These assays have examined various aspects of FB_1_′s biological activity, including its potential to cause cellular damage, disrupt enzyme functions, and interfere with normal cellular communication pathways, further supporting its role in sphingolipid metabolism disruption (EPA, 2025). Despite these advances, complementing research that demonstrates how these findings can translate to practical exposure conditions and further help regulatory decision-making has yet to be released.

## 5 Conclusion

Our research indicates that the current 60 mg/kg FUM GL for cattle remains supported by the available scientific data. The limitations of *meta*-analysis and BMD modeling indicate the need for more comprehensive studies and refined modeling options that address inconsistencies in experimental designs and endpoints. Given the potential impacts on both animal welfare and economic outcomes for the agriculture industry, we recommend that future regulatory assessments of FUM GLs are conducted as new and more robust data emerge.

## Funding statement

This work was financed by the Anderson’s Research Grant, administered through the Ohio State University NC-213 program [Award number: 560900AA24].

## CRediT authorship contribution statement

**Ashli A. Brown:** Conceptualization, Methodology, Formal analysis, Data curation. **Tim Herrman:** Supervision, Writing – review & editing.

## Declaration of competing interest

The authors declare that they have no known competing financial interests or personal relationships that could have appeared to influence the work reported in this paper.

## Data Availability

Data will be made available on request.
